# More Active Intestinal Immunity Developed by Obese Mice Than Non-Obese Mice After Challenged by *Escherichia coli*

**DOI:** 10.3389/fvets.2022.851226

**Published:** 2022-06-03

**Authors:** Dongjie Cai, Bin Tian, Shuang Liang, Yao Cen, Jing Fang, Xiaoping Ma, Zhijun Zhong, Zhihua Ren, Liuhong Shen, Liping Gou, Ya Wang, Zhicai Zuo

**Affiliations:** ^1^College of Animal Science and Technology, Sichuan Agricultural University, Chengdu, China; ^2^Institute of Preventive Veterinary Medicine, Sichuan Agricultural University, Chengdu, China; ^3^Key Laboratory of Animal Disease and Human Health of Sichuan, College of Veterinary Medicine, Sichuan Agricultural University, Chengdu, China; ^4^Animal Nutrition Institute, Sichuan Agricultural University, Chengdu, China

**Keywords:** obesity, mucosal immunity, intestine, *Escherichia coli*, lymphocyte

## Abstract

Obese mice presented lower mortality to non-fatal pneumonia induced by *Escherichia coli* (*E. coli*) than the non-obese mice. However, it remained obscure whether the intestine contributed to the protective effect of obese mice with infection. The 64 non-obese (NOB) mice were divided into NOB-uninfected and NOB-*E. coli* groups, while 64 high-fat diet-induced obesity (DIO) mice were divided into DIO-uninfected and DIO-*E. coli* groups. Mice in *E. coli* groups were intranasally instilled with 40 μl *E. coli* (4.0 ×10^9^ colony-forming units [CFUs]), while uninfected groups with the same volume of phosphate buffer saline (PBS). The T subsets of Intraepithelial lymphocytes (IELs) and lamina propria lymphocytes (LPLs) in the intestine were collected for flow cytometry analysis at 0, 12, 24, and 72 h post-infection, also the duodenum and colon were harvested to survey histopathological change. The results showed that the percentage of CD3^+^T cells in LPLs in DIO-*E. coli* group was significantly lower than that in the DIO-uninfected group after infection (*p* < 0.05). The percentage of CD4^+^T cells in IELs in NOB-*E. coli* was significantly lower than that in DIO-*E. coli* after infection (*p* < 0.05). The percentage of CD8^+^T cells in LPLs in NOB-*E. coli* was significantly lower than that in DIO-*E. coli* at 12 and 24 h (*p* < 0.05). The immunoglobulin A (IgA)^+^ cells in DIO-uninfected were higher than that in NOB-uninfected at all time points (*p* < 0.05). The IgA^+^ cells in DIO-*E. coli* were higher than that in DIO-uninfected at 12, 24, and 72 h (*p* < 0.05). The results revealed that the level of intestinal mucosal immunity in obese mice was more active than that in non-obese mice.

## Introduction

Obesity is a chronic metabolic disease defined as pathologically increased fat mass, which is correlated with diabetes, atherosclerosis, and cardiovascular disease (1–3). Obesity has become a fast-growing global health problem in today's society, which can have a major negative impact on life. There is evidence that obesity affects the immune system (4–6), and an association between obesity and chronic inflammation has been demonstrated (7–9). Studies indicate increased morbidity and mortality in obese individuals following Coronavirus Disease-19 (COVID-19) and influenza infections and increased susceptibility to bacterial infections (10, 11). On the other hand, a study reported that obese mice exhibited more lymphocytes in the bronchoalveolar lavage fluid than normal mice in the case of non-fatal pneumonia that is caused by *Escherichia coli* (*E. coli*) (12). Furthermore, the diet-induced obesity (DIO) mice that are inoculated with *E. coli* presented a delayed pulmonary inflammatory response (13). In addition, the previous study found that the changes of hepatic histopathological damage showed to be less severe in the DIO mice than that in the normal mice following *E. coli* infection (14). Furthermore, the study performed by Wan et al. reported that the mortality of the non-fatal pneumonia of obese mice induced by *E. coli* is lower than that of normal mice (12). Gu et al. reported that obese mice showed increased splenic oxidation and inflammatory levels, while enhanced antioxidant capacity and reduced cytokine levels of the spleen in mice to resist splenic injury after *E. coli* infection (15). However, the effect on intestinal mucosal immunity is unclear at present.

It is shown that obesity has substantial influences on immune surveillance (16), while it is not clear what the role of obesity plays on intestine immunity. The intestine is not only a site of nutrient digestion and microbiota colonization but also a reservoir of immune cells (17). The sizable population of lymphocytes residing in the intestine is far bigger than that in marrow, thymus, spleen, and lymph nodes, which makes the gut a vital immune organ of the body. Moreover, emerging data indicate a link between the intestine and the lung (18, 19), which implies that “intestine-lung” is important for the body against alien microorganisms.

To investigate whether the intestine contributed to the protective effect of obese mice that suffered from *E. coli* infection by nasal inoculation, the intestine T subsets, the lymphocyte, IgA^+^ cells, and goblet cells in the DIO mice challenged by *E. coli* were determined. The information from this research would provide a reference for further study on the interaction between intestine immunity and obesity. Potentially, our findings may lead clinicians or overweight individuals to be more optimistic about obesity.

## Materials and Methods

### Ethics Statement

This study was submitted to and approved by the Animal Welfare Committee of Sichuan Agricultural University (approval number SYXK2019-187). The samples were collected and handled in accordance with the good animal practices required by the Animal Ethics Procedures and Guidelines of the People's Republic of China. Mice used in this study were handled in strict accordance with the guidelines of the Animal Welfare Committee of Sichuan Agricultural University. Mice were euthanized using sodium phenobarbital according to American Veterinary Medical Association guidelines, and every effort was made to minimize animal suffering.

### Animals and Experimental Design

Twenty one-day-old male-specific-pathogen-free Kunming (KM) mice were purchased from the Dossy Animal Center (Chengdu, China). Mice were maintained in individual cages in a specific pathogen-free environment with an automatically controlled 12 h light/dark cycle and free access to food and water. All mice (*n* = 170) were fed for 4 days in advance, and then they were randomly divided into two groups, one of which was fed with a normal diet (*n* = 70), and the other was fed with high-fat diet (*n* = 100). The breeding conditions were as follows: per 7–8 mice lived in one cage; temperature and humidity were maintained at 22 ± 2°C and 55 ± 5%; illumination time was kept natural; food and water were supplied unlimitedly. Mice (*n* = 64) with an obese index exceeding 20% were selected from the high-fat diet group as DIO mice as described previously (15). Then, the normal mice (*n* = 64) were averagely divided into two groups, with 32 mice in each group. The DIO mice were grouped in the same way as normal mice.

### Establishment of Mice Pneumonia Model Induced by *E. coli*

*Escherichia coli* obtained from the Veterinary Medical Laboratory of Sichuan Agricultural University was cultured in Luria-Bertani broth for 18 h at 37°C. Then, the cultured bacteria were centrifuged, and bacterial pellets were resuspended in PBS to produce the inoculums. After reared for 8 weeks, mice in NOB-*E. coli* and DIO-*E. coli* groups were anesthetized by inhalation with moderate ether and challenged intranasally with 40 μl of a bacterial suspension containing 5.9 × 10^10^ colony-forming units (CFUs) *E. coli*; at the same time, the equal volume of PBS was intranasally administered in mice of NOB-uninfected and DIO-uninfected groups.

### Detection of Intestinal T Lymphocyte Subsets by Flow Cytometry

The mice were euthanized with ether overdose, and then the whole intestine was taken from each mouse. Briefly, mesentery was removed from the intestine and the intestinal tract was longitudinally cut and then placed in D-Hank on standby. The sample was incubated in 5 ml of trypsin-ethylenediaminetetraacetic acid (EDTA) solution that contains 0.25% trypsin and 0.02% EDTA for 40 min in a water bath shaker at 37°C. The solution was filtered with a sterile 48-μm-pore metal sieve, then centrifuged at 1,000 r/min for 5 min, the supernatants were discarded. The cell pellets were resuspended with 2.7 ml of 1640 medium that contains 5% newborn calf serum (NCS), and then 1.8 ml of 100% percoll solution was added, which were fully mixed to make a 40% percoll suspension. Then 1 ml of 70% percoll suspension was added gently to make a percoll gradient separation solution, centrifuged at 600 r/min for 20 min, and the cells were further purified and collected from the interface between 40% and 70% percoll layers, then the cells were washed once with 1640 medium that contains 5% NCS, which were then purified intestinal epithelium (IELs). At the same time, the intestine samples were dealt with trypsin-EDTA solution in 5 ml of Roswell Park Memorial Institute Medium (RPMI) 1640 medium that contains 0.1% collagenase II for 40 min at 37°C, and subsequent steps as above were repeated to separate lamina propria lymphocytes (LPLs). Cells were counted under a microscope, and viability was tested by trypan blue exclusion. Cells with viability of more than 90% were used for the next step. In each reaction, 1 × 10^6^ cells were used for staining. IELs and LPLs were stained with the following antibodies: fluorescein isothiocyanate (FITC) Hamster Anti-Mouse CD3e, PE Rat Anti-Mouse CD8α, and PerCP Rat Anti-Mouse CD4. All monoclonal antibodies were purchased from BD Biosciences (USA). Then T lymphocyte subsets were analyzed using a FACScan flow cytometer (Becton-Dickinson, San Diego, CA, USA) and CellQuest software (Becton-Dickinson, Mountain View, CA, USA). For data analysis, FlowJo software V7.6 (Tree Star) was used.

### Intestinal Histological Examination

Samples from the duodenum and colon were fixed in 4% paraformaldehyde for 48 h at room temperature, dehydrated through graded alcohol to xylene, and paraffin-embedded at 60°C for 3 h. Subsequently, 5 μm sections were prepared (Reichert-Jung Ultramicrotome Leica RM 206). Part of the intestinal tissue sections was stained with hematoxylin and eosin (H&E) (Solarbio) for histopathological changes. Images were captured using a microscope (Olympus, Tokyo, Japan). Eight disconnected slices were randomly selected at each time point, and 5 fields of view were selected for each slice for counting, and cells were normalized to field area (mm^2^). Mechanical Manual Cell Counter (YAMI, China) was used for cell counting. All evaluations were performed by two pathologists who were blinded to the treatment regimens.

### Immunohistochemistry

Immunohistochemical staining was performed with an SABC-POD Kit (Boster Biological Technology Co. Ltd.) according to the instructions of the manufacturer. Briefly, paraffin-embedded tissue sections were deparaffinized, rehydrated, and quenched with 3% hydrogen peroxide solution for 15 min at room temperature. The slides were pretreated by heating the slides for 25 min in 10 mM citrate buffer. After they had been washed with PBS, the slides were blocked with goat serum for 30 min. Rabbit Anti-Mouse IgA (GENXPAN) was used as the primary antibody, horseradish peroxidase (HRP) Goat Anti-Rabbit IgG (TransGen Biotech) was used as the secondary antibody. Tissue staining was visualized with 3,3′-diaminobenzidine (DAB). Images were captured using a microscope (Olympus, Tokyo, Japan). Eight disconnected slices were randomly selected at each time point, and 5 fields of view were selected for each slice for counting, and cells were normalized to field area (mm^2^). Mechanical Manual Cell Counter (YAMI, China) was used for cell counting. All evaluations were performed by two pathologists who were blinded to the treatment regimens.

### Alcian Blue and Periodic Acid-Schiff (AB-PAS) Staining

After deparaffinization and rehydration, the prepared sections were treated with alcian blue dye (Solarbio) for 10 min, washed with running tap water for 2 min, and stained with periodic acid (Solarbio) for 5 min. The slices were treated with Schiff's staining (Solarbio) for 10 min under protection from light. After washing with the tap running water, the sections were subjected to Scott's blue staining for 30 s, followed by dehydrated through xylene and coverslipped. Images were captured using a microscope (Olympus, Tokyo, Japan). Eight disconnected slices were randomly selected at each time point, and 5 fields of view were selected for each slice for counting, and cells were normalized to field area (mm^2^). Mechanical Manual Cell Counter (YAMI, China) was used for cell counting. All evaluations were performed by two pathologists who were blinded to the treatment regimens.

### Statistical Analysis

Statistical analysis was performed using the SPSS 17 statistical software (IBMCorp., Armonk, NY, USA). Statistical significance of differences was tested using one-way ANOVA [least significant difference (LSD) or Dunnett's T3] between groups. Data are presented as the mean ± standard deviation (SD). Values of *p*: a, *p* < 0.05 compared with group NOB-uninfected; A, *p* < 0.01 compared with group NOB-uninfected; b, *p* < 0.05 compared with group DIO-*E. coli*; B, *p* < 0.01 compared with group DIO-*E. coli*. All experiments were performed in triplicate.

## Results

### Clinical Symptoms of Mice Following *E. coli* Challenge

At 0 h (untreated), the mice in each group had a good mental state, sensitive reaction, and normal drinking and appetite. There were no significant changes in the mental status, feeding status, and activity status of the mice intranasally instilled with PBS. While the mice treated with *E. coli*, especially at 12 h, had symptoms of poor mental status, decreased or stopped feeding, and immobility. No death occurred in all groups.

### Effects of Obesity on T Lymphocyte Subsets in Intestinal IELs and LPLs of Mice Challenged With *E. coli*

**Figure 1:**
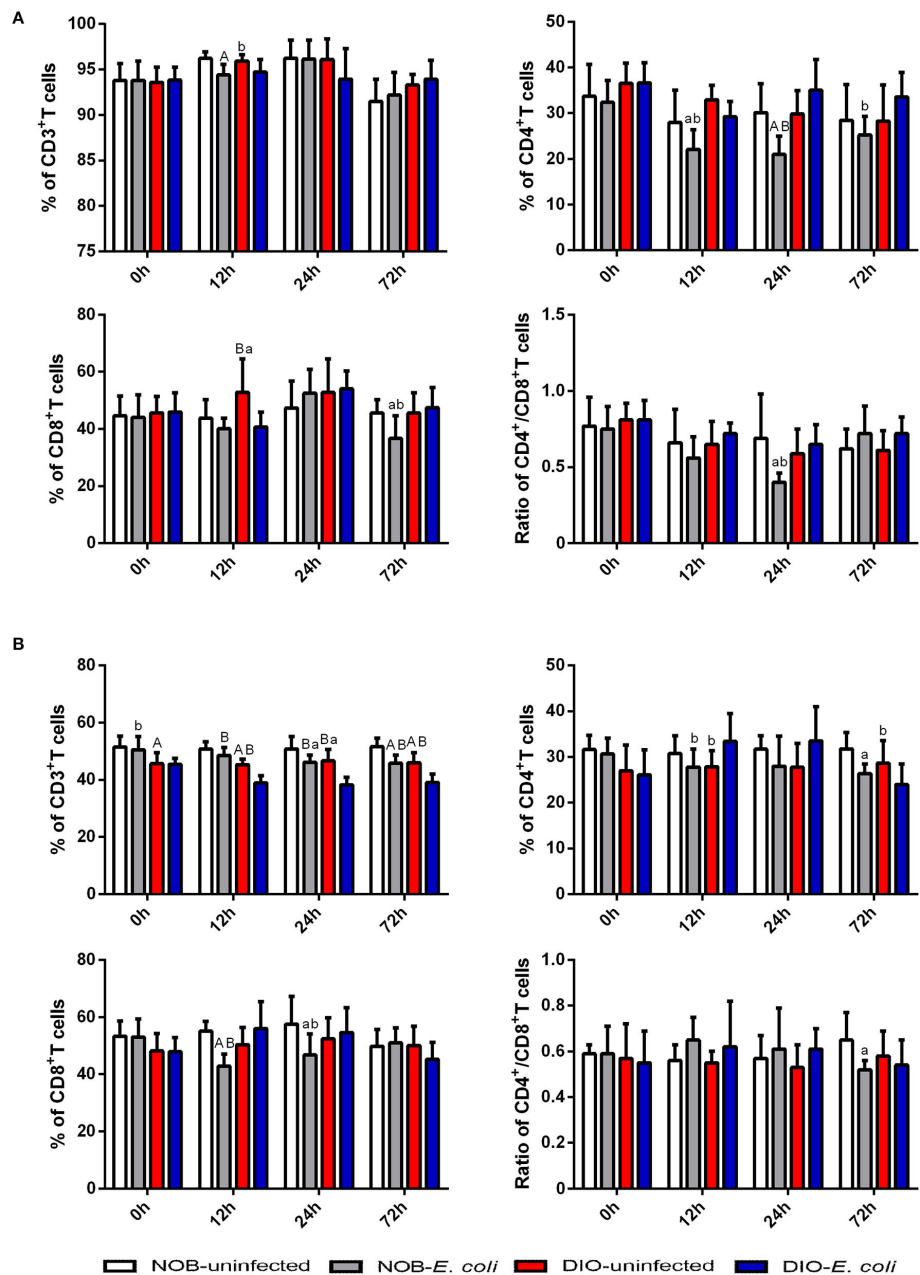
The alterations of intestinal CD3^+^T cells, CD4^+^T cells, CD8^+^T cells, and CD4^+^/CD8^+^T cells in 0, 12, 24, and 72 h post-infection. Intestines were collected aseptically for lymphocyte isolation from mice of each group in 0, 12, 24, and 72 h post-infection. **(A)** The percentage of CD3^+^T cells, CD4^+^T cells, CD8^+^T cells, and CD4^+^/CD8^+^T cells in the intestinal epithelium (IELs). **(B)** The percentage of CD3^+^T cells, CD4^+^T cells, CD8^+^T cells, and CD4^+^/CD8^+^T cells in intestinal lamina propria (LPLs). The data from each group are presented as the mean ± SD (*n* = 8). a, *p* < 0.05 compared with group NOB-uninfected; A, *p* < 0.01 compared with group NOB-uninfected; b, *p* < 0.05 compared with group DIO-*E. coli*; B, *p* < 0.01 compared with group DIO-*E. coli*.

In Figure 1A, there is no significant difference in the percentage of CD3^+^ and CD4^+^T cells and the ratio of CD4^+^/CD8^+^T cells in intestinal IELs of mice between groups DIO-uninfected and NOB-uninfected (*p* > 0.05; Supplementary Figure S1A). The percentage of CD8^+^T cells in mice of group DIO-uninfected was significantly higher than that in group NOB-uninfected at 12 h post-infection (*p* < 0.05). The percentage of CD3^+^T cells in mice of group NOB-*E. coli* was significantly lower than that in group NOB-uninfected at 12 h post-infection (*p* < 0.01), the percentage of CD4^+^T cells in group NOB-*E. coli* was significantly lower than that in group NOB-uninfected at 12 and 24 h (*p* < 0.05), and the percentage of CD8^+^T cells in group NOB-*E. coli* was significantly lower than that of group NOB-uninfected at 72 h (*p* < 0.05), and the ratio of CD4^+^/CD8^+^T cells in group NOB-*E. coli* was significantly lower than that of group NOB-uninfected at 24 h (*p* < 0.05). The percentage of CD3^+^T cells in mice of group DIO-*E. coli* was significantly lower than that in group DIO-uninfected at 12 h (*p* < 0.05), and the percentage of CD8^+^T cells in group DIO-*E. coli* was significantly lower than that in group DIO-uninfected at 12 h (*p* < 0.01). After infection, the percentage of CD4^+^T cells and the ratio of CD4^+^/CD8^+^T cells in group DIO-*E. coli* were not significantly different from that of group DIO-uninfected (*p* > 0.05). There was no significant difference in the percentage of CD3^+^T cells in mice of group DIO-*E. coli* when compared with group NOB-*E. coli* at each time point (*p* > 0.05), and the percentage of CD4^+^T cells in group DIO-*E. coli* was significantly higher than that in group NOB-*E. coli* at 12, 24, and 72 h (*p* < 0.05), the ratio of CD8^+^T cells in group DIO-*E. coli* was significantly higher than that in group NOB-*E. coli* at 72 h (*p* < 0.05), and the ratio of CD4^+^/CD8^+^T cells in group DIO-*E. coli* was significantly higher than that in group NOB-*E. coli* at 24 h (*p* < 0.05).

In Figure 1B, there is no significant difference in the percentage of CD4^+^ and CD8^+^T cells and the ratio of CD4^+^/CD8^+^T cells in the intestinal LPLs of mice between groups DIO-uninfected and NOB-uninfected (*p* > 0.05). The percentage of CD3^+^T cells in group DIO-uninfected was significantly lower than that in group NOB-uninfected at each time point (*p* < 0.05; Supplementary Figure S1B). The percentage of CD3^+^T cells in mice of group NOB-*E. coli* was significantly lower than group NOB-uninfected at 24 and 72 h post-infection (*p* < 0.05), the percentage of CD4^+^T cells in group NOB-*E. coli* was significantly lower than that in group NOB-uninfected at 72 h (*p* < 0.05), the percentage of CD8^+^T cells in group NOB-*E. coli* was significantly lower than that in NOB-uninfected at 12 and 24 h (*p* < 0.05), and the ratio of CD4^+^/CD8^+^T cells in group NOB-*E. coli* was significantly lower than that in group NOB-uninfected at 72 h (*p* < 0.05). There was no significant difference in the percentage of CD8^+^T cells and the ratio of CD4^+^/CD8^+^T cells in mice between groups DIO-*E. coli* and DIO-uninfected (*p* > 0.05), the percentage of CD3^+^T cells in group DIO-*E. coli* was significantly lower than that in group DIO-uninfected at 12, 24, and 72 h post-infection (*p* < 0.01), the percentage of CD4^+^T cells in group DIO-*E. coli* was significantly higher than that in group DIO-uninfected at 12 h (*p* < 0.05), while significantly lower than that in group DIO-uninfected at 72 h (*p* < 0.05). The percentage of CD3^+^T cells in mice of group NOB-*E. coli* was significantly higher than that in group DIO-*E. coli* at each time point (*p* < 0.05), the percentage of CD4^+^T cells in group NOB-*E. coli* was significantly lower than that in group DIO-*E. coli* at 12 h (*p* < 0.05), and the percentage of CD8^+^T cells in group NOB-*E. coli* was significantly lower than that of group DIO-*E. coli* at 12 and 24 h (*p* < 0.05), and the ratio of CD4^+^/CD8^+^T cells in group NOB-*E. coli* was not significantly different from group DIO-*E. coli* at each time point (*p* > 0.05).

### Effects of Obesity on IELs in Duodenum of Mice Challenged With *E. coli*

**Figure 2:**
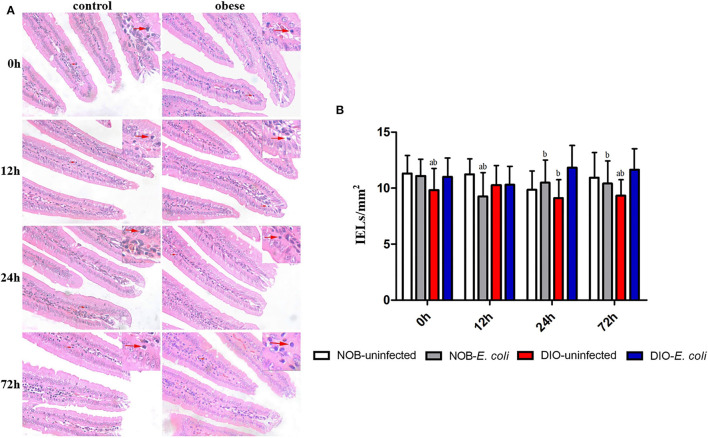
The counting of duodenal intestinal epithelium (IELs) of each group in 0, 12, 24, and 72 h post-infection. **(A)** Representative photomicrographs of H&E-stained duodenum of mice in control (NOB-*E. coli*) and obese (DIO-*E. coli*) group. 0, 12, 24, and 72 h were the time points after the mice were nasally instilled with *E. coli*. Images were taken at 400 × magnification. *n* = 8 mice per group. The red arrow points to the lymphocytes in the duodenal epithelium. **(B)** Statistical analysis of duodenal IELs counts per mm^2^ in H&E-stained sections of each group in 0, 12, 24, and 72 h post-infection. a, *p* < 0.05 compared with group NOB-uninfected; A, *p* < 0.01 compared with group NOB-uninfected; b, *p* < 0.05 compared with group DIO-*E. coli*; B, *p* < 0.01 compared with group DIO-*E. coli*.

In Figure 2A, the duodenum tissue sections show that the edges of the villi were smooth, the shape and the size of crypts were normal, and villus atrophy, crypt abscesses, and hyperplasia of crypt cell epithelium were absent at each time point. As shown in Figure 2B, the number of IELs in the duodenum of mice in group DIO-uninfected is significantly lower than that in group NOB-uninfected at 0 and 72 h (*p* < 0.05). The number of IELs in group NOB-*E. coli* was significantly lower than that in group NOB-uninfected at 12 h (*p* < 0.05). Compared with group DIO-uninfected, the number of IELs in group DIO-*E. coli* was increased significantly at 24 and 72 h (*p* < 0.05). Compared with group DIO-*E. coli*, the number of IELs in group NOB-*E. coli* was significantly reduced at 12, 24, and 72 h (*p* < 0.05). As shown in Supplementary Figure S2, the treatment of intranasal instillation of *E. coli* did not affect the colon tissue structure of mice in groups NOB-*E. coli* and DIO-*E. coli*. However, the lymphocytes in colon tissue sections were accumulated in LPL at 12, 24, and 72 h post-infection both in NOB-*E. coli* and DIO-*E. coli*.

### Effects of Obesity on IgA^+^ Cells in Duodenum of Mice Challenged With *E. coli*

**Figure 3:**
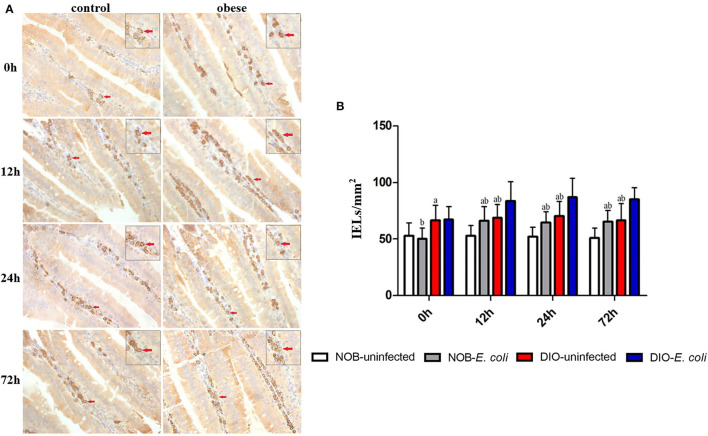
The counting of duodenal IgA^+^ lamina propria (LPLs) of each group in 0, 12, 24, and 72 h post-infection. **(A)** Representative photomicrographs of immunostaining of IgA^+^ cells in the duodenum of mice in control (NOB-*E. coli*) and obese (DIO-*E. coli*) group. 0, 12, 24, and 72 h were the time points after the mice were nasally instilled with *E. coli*. Images were taken at 400 × magnification. *n* = 8 mice per group. The red arrows point to the IgA^+^ cells in the lamina propria of duodenum. The small picture in the upper right corner of each picture is a magnified IgA^+^ cells area. **(B)** Statistical analysis of duodenal IgA^+^ LPLs counts per mm^2^ in immunostaining sections of each group in 0, 12, 24, and 72 h post-infection. a, *p* < 0.05 compared with group NOB-uninfected; A, *p* < 0.01 compared with group NOB-uninfected; b, *p* < 0.05 compared with group DIO-*E. coli*; B, *p* < 0.01 compared with group DIO-*E. coli*.

In Figure 3, the number of IgA^+^ cells in the duodenum of mice in group DIO-uninfected was significantly higher than that in group NOB-uninfected at 0, 12, 24, and 72 h (*p* < 0.05). The number of IgA^+^ cells in group NOB-*E. coli* was significantly higher than that in group NOB-uninfected at 12, 24, and 72 h (*p* < 0.05). The number of IgA^+^ cells in group DIO-*E. coli* was significantly higher than that in group DIO-uninfected at 12, 24, and 72 h (*p* < 0.05). The number of IgA^+^ cells in group NOB-*E. coli* was significantly lower than that in group DIO-*E. coli* at all time points (*p* < 0.05).

### Effects of Obesity on Goblet Cells in Duodenum of Mice Challenged With *E. coli*

**Figure 4:**
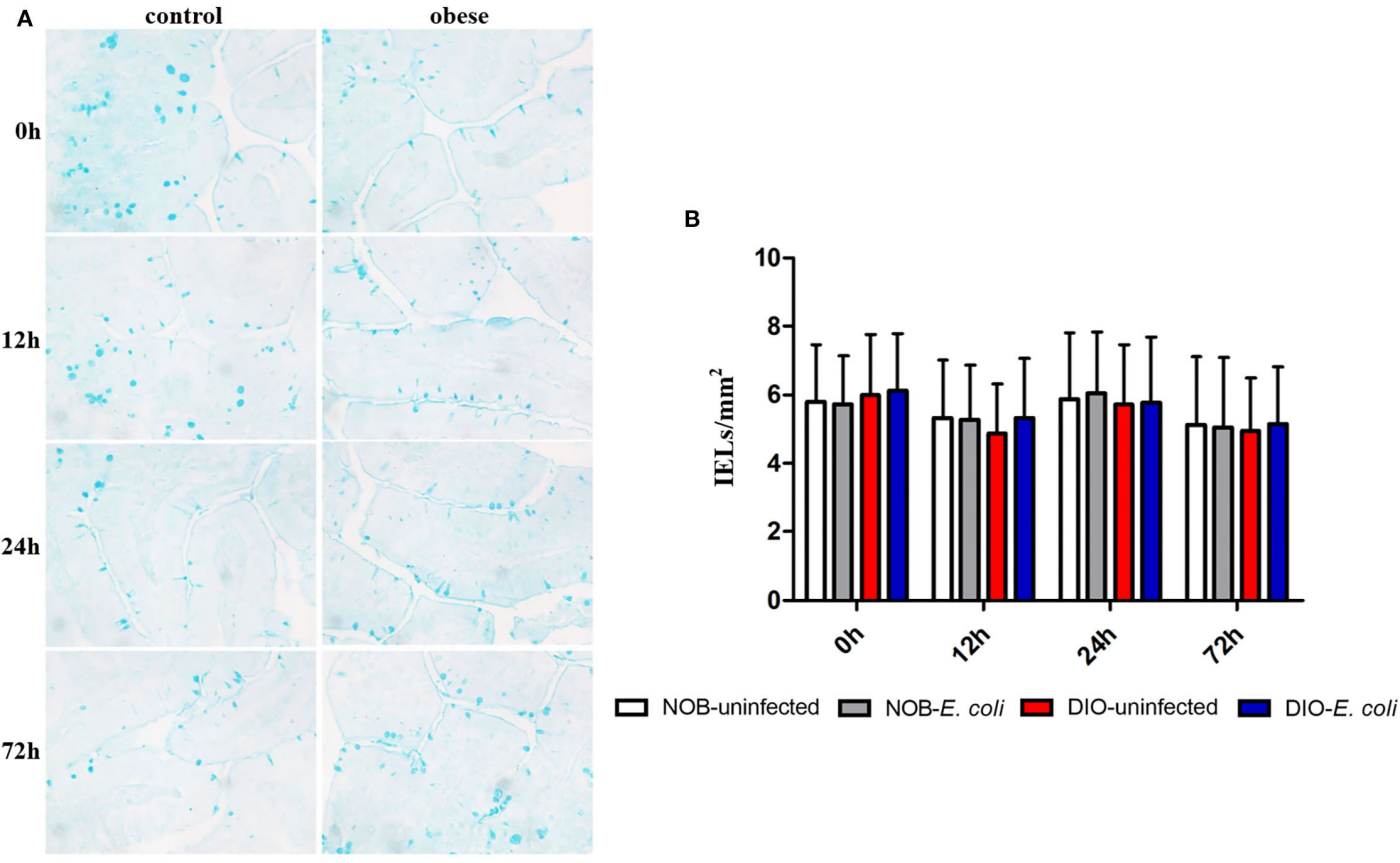
The counting of duodenal goblet cells of each group in 0, 12, 24, and 72 h post-infection. **(A)** Representative photomicrographs of goblet cells stained by alcian blue and periodic acid-Schiff (AB-PAS) in duodenum of mice in control (NOB-*E. coli*) and obese (DIO-*E. coli*) group. 0, 12, 24, and 72 h were the time points after the mice were nasally instilled with *E. coli*. Images were taken at 400 × magnification. *n* = 8 mice per group. The blue dotted areas in the picture are the goblet cells. **(B)** Statistical analysis of duodenal goblet cells counts per mm^2^ in sections stained by AB-PAS of each group in 0, 12, 24, and 72 h post-infection.

In Figure 4 and Supplementary Figure S3, it can be seen that the goblet cells are dark blue after AB-PAS staining, and they are scattered among the absorptive cells of the intestinal villi epithelium. There was no significant difference in the number of goblet cells in the duodenum of mice between groups DIO-uninfected and NOB-uninfected at each time point (*p* > 0.05). There was no significant difference in the number of goblet cells between groups NOB-*E. coli* and DIO-*E. coli* at each time point (*p* > 0.05). At 0 h, the number of goblet cells in the colon of group DIO-uninfected mice was more than that in group NOB-uninfected (Supplementary Figure S3). At 12, 24, and 72 h, the number of goblet cells in group DIO-*E. coli* was more than that in group NOB-*E. coli*. However, there was no difference in the number of goblet cells in groups NOB-*E. coli* and DIO-*E. coli* (*p* > 0.05).

## Discussion

Obesity is increasingly a hot topic for researchers, which is associated with many major diseases, especially pulmonary disease, such as COVID-19 (20, 21). Contrarily, a novel finding published by Paslakis et al. has indicated that adipocytes as the major constituents of the omentum could play an important role in protecting against infection by generating defensins that are natural antimicrobial peptides (22). Another report demonstrated that vertebrate adipocytes could migrate to wounds to undertake several local functions, such as driving wound repair and preventing infections (23). Furthermore, another known function of adipocytes is antimicrobial activities, for example, *Staphylococcus aureus* infection of healthy skin causes rapid proliferation of dermal adipose cells, and impaired adipogenesis results in increased skin infections (24, 25). Although obesity has been identified as a risk factor for adverse outcomes of various diseases, to some extent, which is actually good for the body under some special conditions as the results show in this study.

The key immune system from the intestinal tract is gut-associated lymphoid tissue that is mainly comprised of IELs and LPLs (26, 27). More than 90% of IELs are CD3^+^T cells that are interspersed between epithelial cells along the entire length of the intestine (28), which is in line with the present study. This unique location of IELs in the interface between the lumen and the LPL is beneficial to the integrity of the epithelium. IELs are potent, rapidly activating cytolytic and immunoregulatory effectors that can protect host tissues from infection (17). In the present study, there was no significant change in the population of IELs. The reason why the population of IELs did not show prominently differences is that IELs may rely on a complicated developmental pathway (29). LPLs have been traditionally associated with CD4^+^T cells, which play an important role as effective sites in the adaptive immune system (30). On the other hand, the amount of the LPLs was changed significantly after the mice were challenged by *E. coli*. The percentage of CD3^+^T cells in obese mice (group DIO-uninfected and DIO-*E. coli*) was remarkably decreased than that in non-obese mice (group NOB-uninfected and NOB-*E. coli*). The results showed that obesity could affect the number of LPLs in mice. Moreover, this result illustrated that the *E. coli* infection can trigger a common mucosal immune system to resist *E. coli*, leading to the reduction of the percentage of CD3^+^T cells of LPLs. The reduction of CD3^+^T cells of LPLs can be partially explained by the following: (1) CD3^+^T cells go back to blood circulation and (2) migration of B cells from the blood to the LPL dilutes the proportion of CD3^+^T cells. Lymphocytes of various tissues are always in homeostasis, and they go from blood to tissue and back to blood continuously. The recirculation of lymphocytes is not random, which is regulated by the mechanism termed “lymphocyte homing” (31). Sensitized lymphocytes not only traffic through lymphoid organs but also can access and recirculate through extra lymphoid immune effector sites, such as intestinal LPL and pulmonary interstitium, which is contributed to the “common mucosal immune system” (32, 33). T cells play a vital role in protecting the respiratory mucosa from infection, so the migration of T cells from the intestine to the lung may contribute to the decline of the percentage of CD3^+^T cells. Moreover, the process of lymphocyte homing can be accelerated by diverse inflammatory cytokines, such as interleukin (IL)-1, IL-2, and tumor necrosis factor α (TNF-α) (34). The difference in CD3^+^T cells in LPLs between obesity and normal mice may rely on the different inflammatory status, since the high expressions of inflammatory cytokines stimulate the process of lymphocyte homing in obese mice (15).

*Escherichia coli* infection in this study did not affect the structure of intestines in obese and normal mice. Nevertheless, the increased accumulation of LPLs was observed in both obese and normal mice challenged with *E. coli*. The results were consistent with the counting of IgA^+^ cells in LPL of the duodenum. The number of IgA^+^ cells in the duodenum of both obese and normal mice was increased remarkably after the mice were instilled with *E. coli*, and the number of IgA^+^ cells in obese mice was more than that of normal mice after *E. coli* infection. The whole intestinal tract is covered with a mucus layer, which is a reservoir for secretory immunoglobulin A (sIgA) (35). sIgA, a protein derived from IgA^+^ plasma cell, is regarded as a benign antibody in that it fails to bind complement (which would elicit an inflammatory response) and functions mainly as an inhibitor of pathogenic attachment to the underlying epithelium (36). Obese mice owned more IgA^+^ cells of LPL in the duodenum, which contributed to more strong intestinal mucosal immunity and provided some clues to account for the protective role of obesity. Although there was no difference in the number of goblet cells in obese and normal mice, the number of goblet cells of the colon in obese mice was more than that in normal mice after being challenged by *E. coli*, illustrating that obese mice had a better immune capacity to fight infection than normal mice.

In this study, pathogen-free KM mice were used to analyze the role of obesity in *E. coli* infection. Pathogen-free mice are extremely sensitive to microbial infection because they have been living in a sterile environment, which has led to its widespread use in disease models, including obesity (37, 38). If non-pathogen-free mice are used, this test may yield two outcomes. One result is that the modeling will be unsuccessful, because the normal-grade mice have rich intestinal flora and natural resistance to *E. coli*, so that the mice are not infected with *E. coli*. Another possible response would be to cause insignificant differences in results, as the number of IgA^+^ plasma cells and T cells in intestine non-pathogen-free mice is significantly higher than that in pathogen-free mice (39–41). This means that even if the use of highly pathogenic *E. coli* or increasing the dose results in successful infection of normal-grade mice, the difference in results will not be significant due to the high basic number of intestinal immune cells. Therefore, the use of pathogen-free mice to explore infectious diseases in this study is scientific. The test using pathogen-free mice can not only eliminate the interference of various microorganisms on results but also improve the accuracy of results.

In conclusion, *E. coli* infection can bring about the reduction of the percentage of CD3^+^T cells and increment of IgA^+^ cells in LPL of the duodenum, and the intestinal mucosal immunity in obese mice was more active than those in normal mice after being challenged by *E. coli* in this study. Obesity showed a favorable effect on the body in the state of *E. coli* infection, although it is associated with an increased risk of pneumonia in mice. Therefore, obesity is a double-edged sword for the body. Since we only studied the changes between 0 and 72 h after non-fatal *E. coli* infection of mice, we plan to use different strains and prolong the infection time to further study the role of obesity in bacterial infection. We hope that in the near future, we might be able to improve our knowledge of obesity biology and treat obesity without bias.

## Data Availability Statement

The original data presented in the study are included in the article/Supplementary Material, further inquiries can be directed to the corresponding author.

## Ethics Statement

The animal study was reviewed and approved by Animal Welfare Committee of Sichuan Agricultural University (Approval Number SYXK2019-187).

## Author Contributions

DC and SL performed the experiments and drafted the manuscript. BT and YC analyzed the data. JF, XM, ZCZ, and ZR developed the article concept and designed the manuscript. LS, LG, YW, and ZJZ supervised and revised the manuscript. All authors contributed to the article and approved the submitted version.

## Funding

This study was supported by the China Agriculture (Beef Cattle/Yak) Research System (CARS-37) and the Sichuan beef cattle innovation team of the National Modern Agricultural Industry Technology System (SCCXTD-2020-13).

## Conflict of Interest

The authors declare that the research was conducted in the absence of any commercial or financial relationships that could be construed as a potential conflict of interest.

## Publisher's Note

All claims expressed in this article are solely those of the authors and do not necessarily represent those of their affiliated organizations, or those of the publisher, the editors and the reviewers. Any product that may be evaluated in this article, or claim that may be made by its manufacturer, is not guaranteed or endorsed by the publisher.
