# Supplementary material for: More Active Intestinal Immunity Developed by Obese Mice Than Non-Obese Mice After Challenged by Escherichia coli
Source: Front Vet Sci. 2022 Jun 3;9:851226. doi: 10.3389/fvets.2022.851226 (PMC9205201; doi:10.3389/fvets.2022.851226)

Supplementary Material

# Supplementary Figures

**
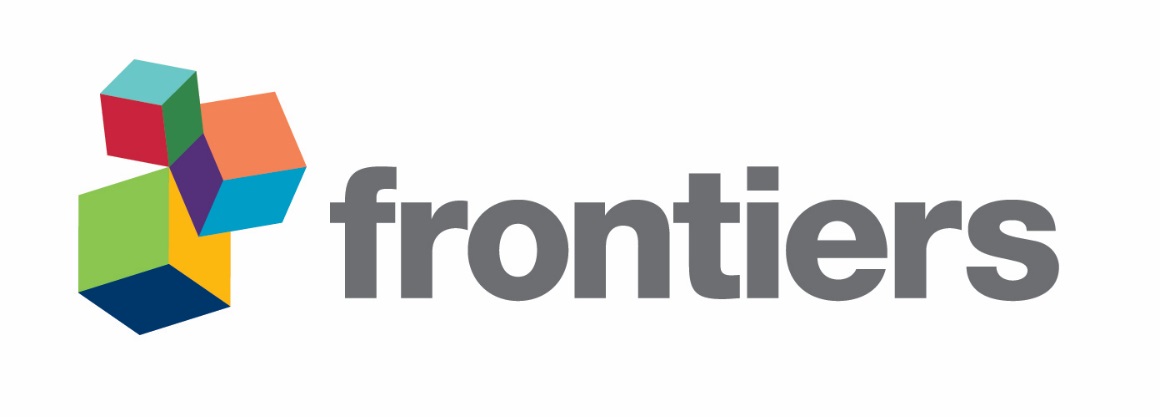
**

**Supplementary Figure 1SA. The analysis of CD3^+^ T cell in intestinal intraepithelial lymphocytes by flow cytometry.** control: NOB-*E. coli* group, obese: DIO-*E. coli* group. 0 h, 12 h, 24 h and 72 h were the time points after the mice nasally instilled with *E. coli*.


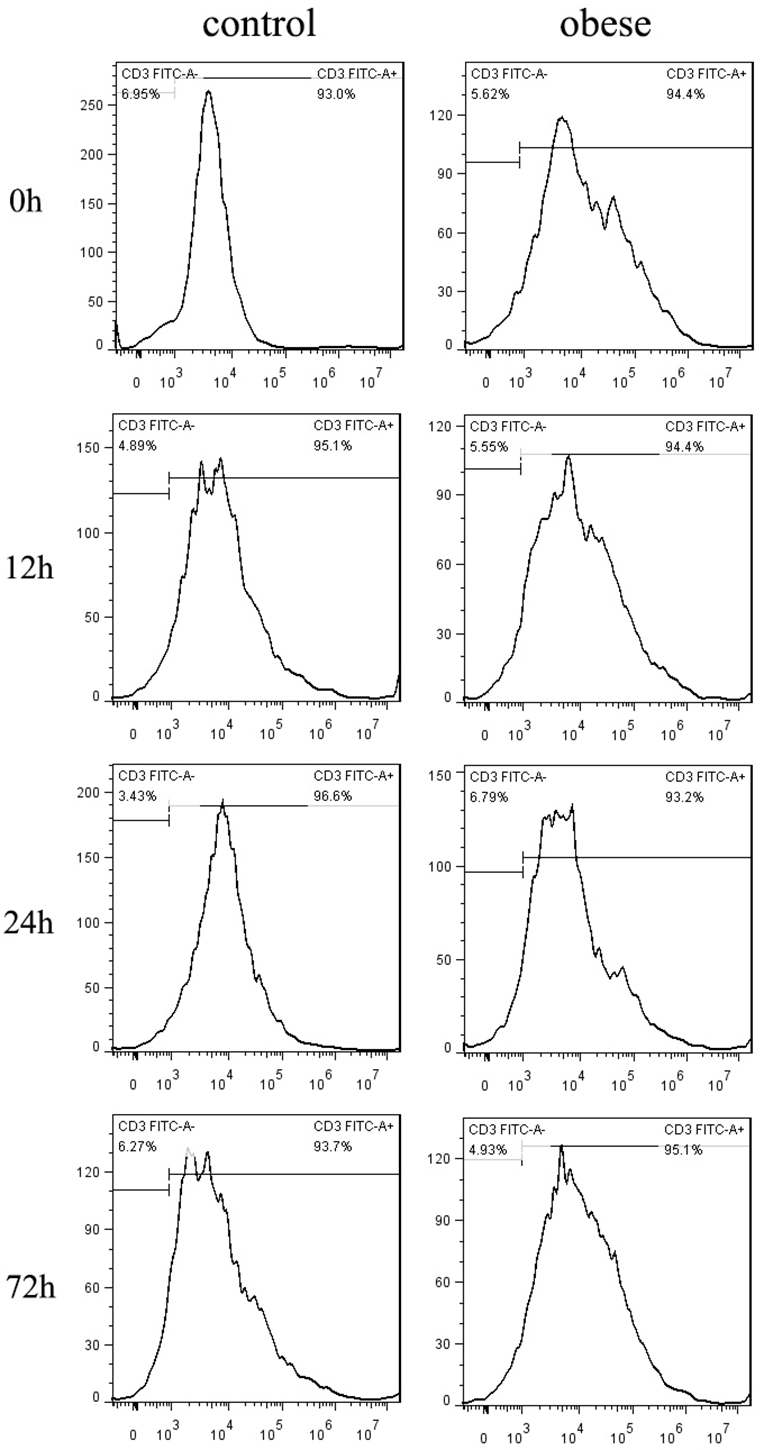


**Supplementary Figure 1SB. The analysis of CD3^+^T cell in intestinal lamina propria lymphocytes by flow cytometry.** control: NOB-*E. coli* group, obese: DIO-*E. coli* group. 0 h, 12 h, 24 h and 72 h were the time points after the mice nasally instilled with *E. coli*.


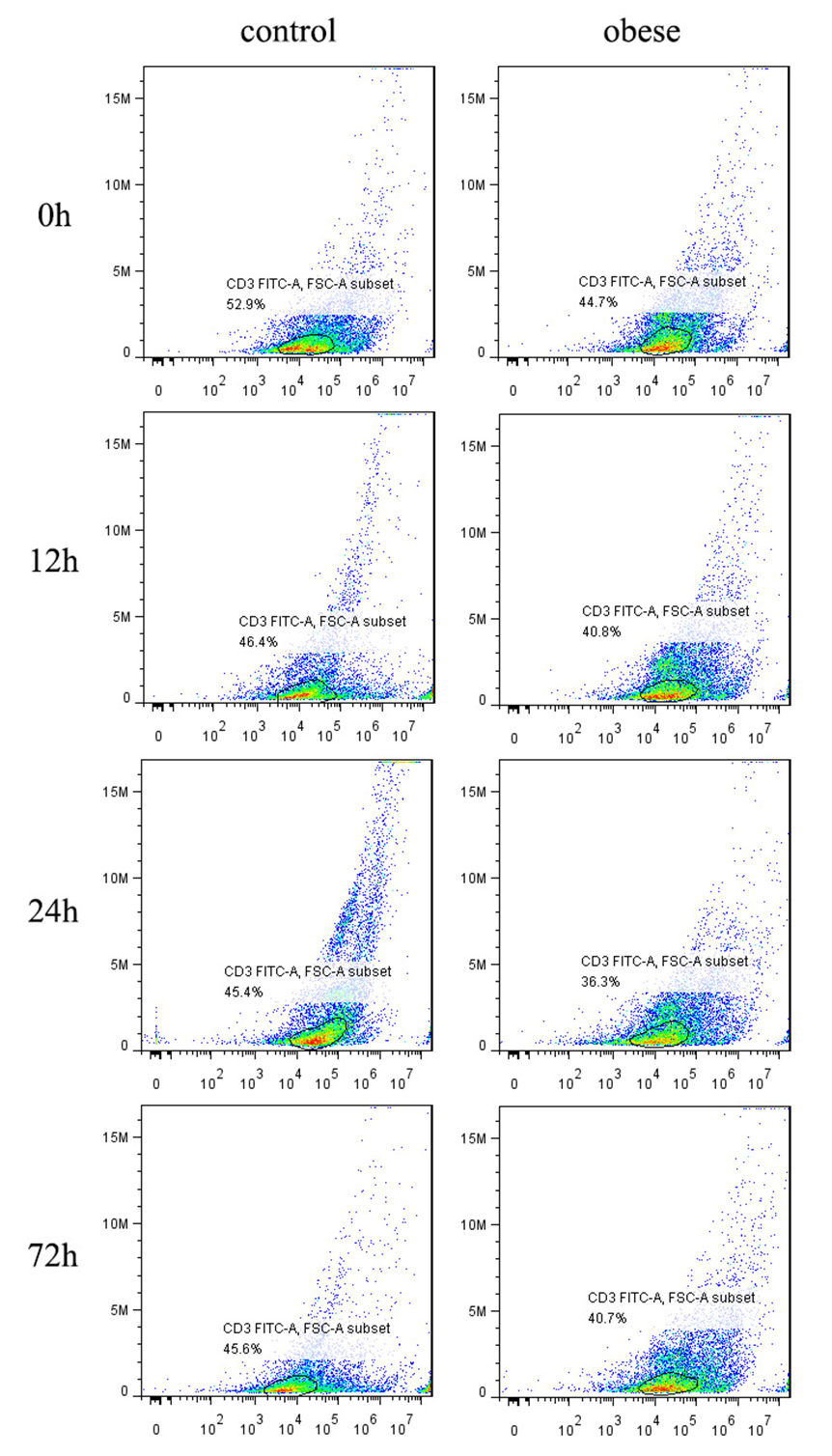


**Supplementary Figure 2S. The histomorphology of the colon of control (**NOB-*E. coli***) and obese (**DIO-*E. coli***) group in 0 h, 12 h, 24 h and 72 h post infection.** The red circle in the figure shows the increased accumulation of lymphocytes was observed in colon. Images were taken at 400× magnification.


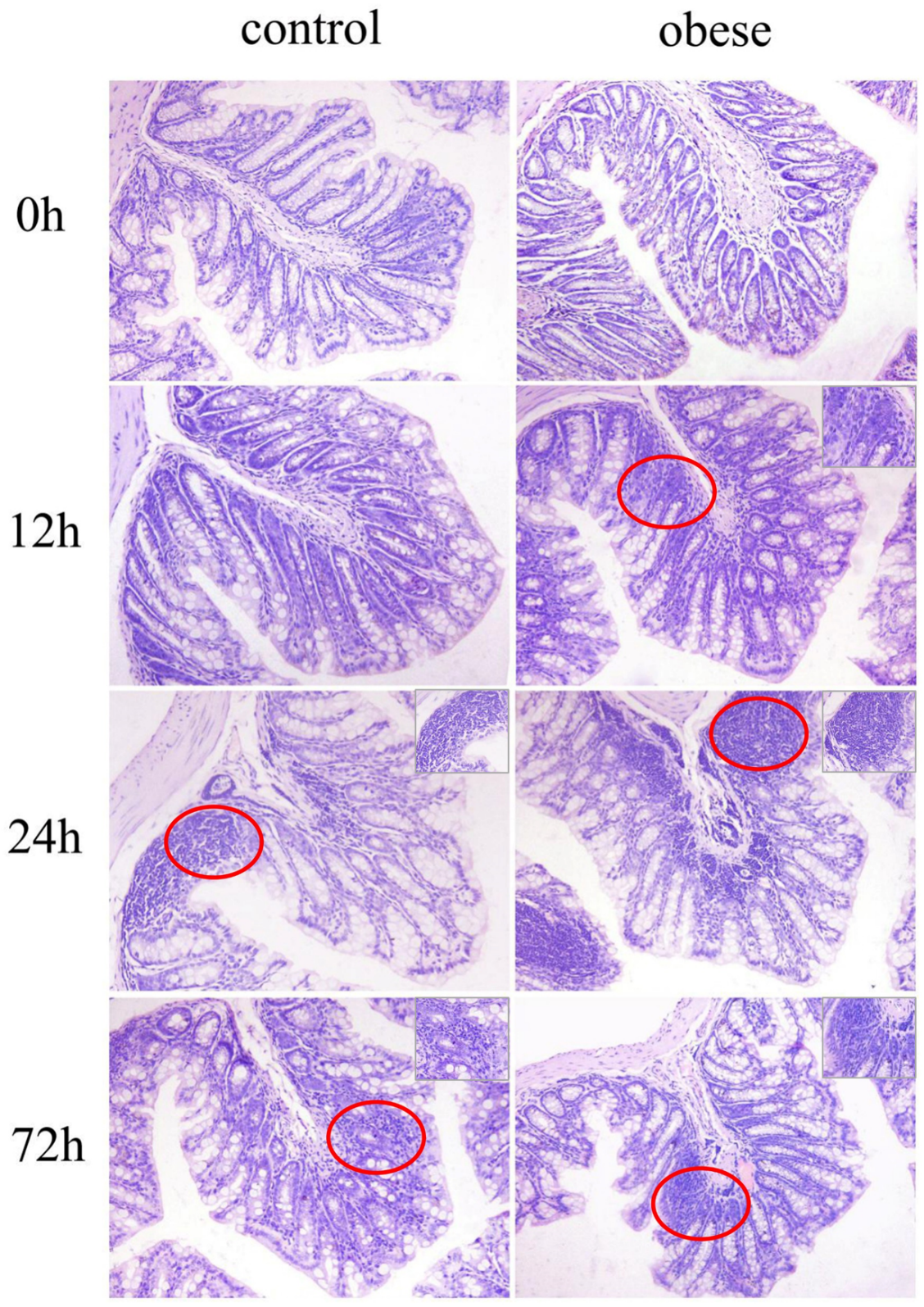


**Supplementary Figure 3S. The goblet cells stained by AB-PAS in the colon of control (**NOB-*E. coli***) and obese (**DIO-*E. coli***) group in 0 h, 12 h, 24 h and 72 h post infection.** The blue dotted areas in the picture are the goblet cells in colon. Images were taken at 400× magnification.


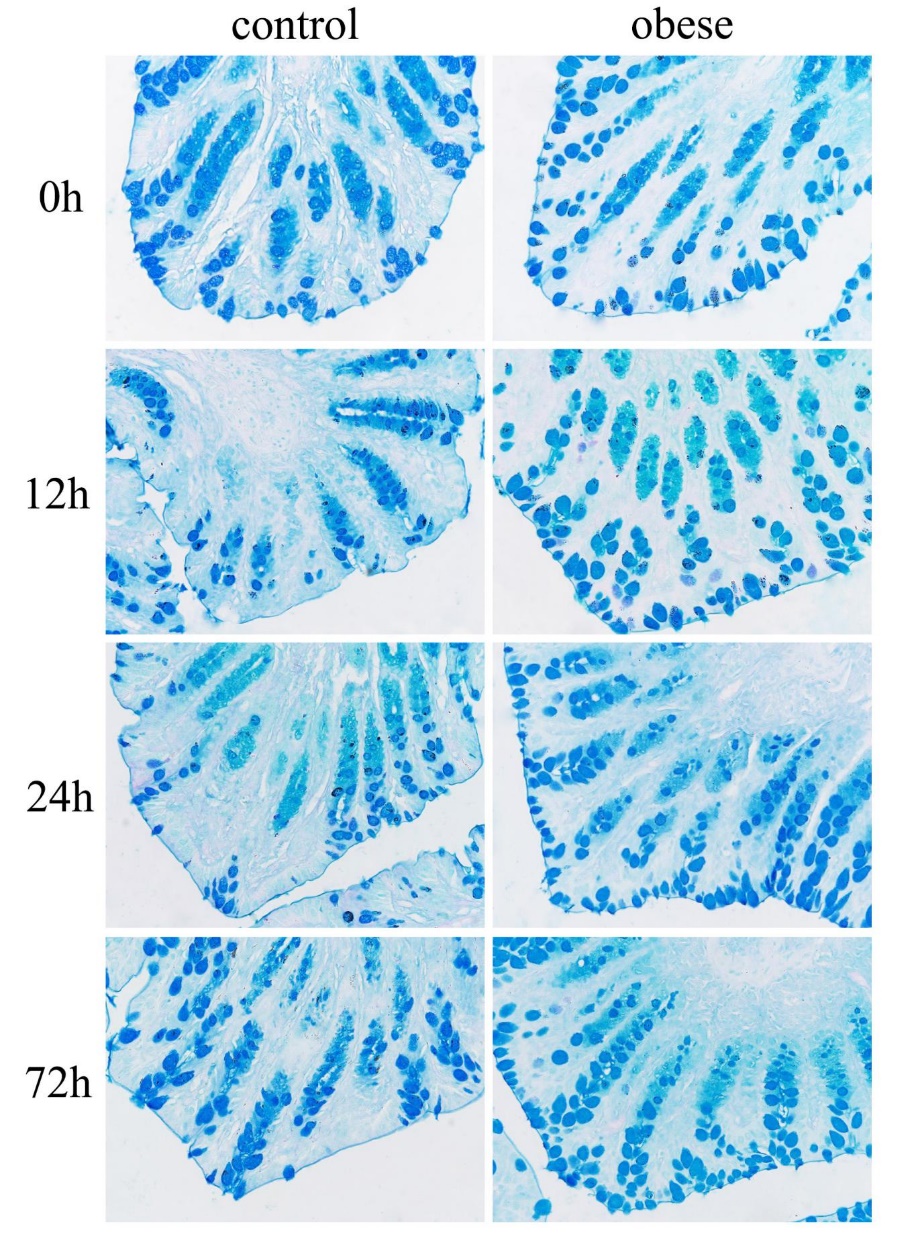

Supplement: Supplementary file 1 [file Data_Sheet_1.DOCX]
